# The Novel Dipeptide Translocator Protein Ligand, Referred to As GD-23, Exerts Anxiolytic and Nootropic Activities

**Published:** 2015

**Authors:** P. Yu. Povarnina, S. A. Yarkov, T. A. Gudasheva, M. A. Yarkova, S. B. Seredenin

**Affiliations:** V.V. Zakusov Research Institute of Pharmacology of RAMS, ul. Baltiyskaya, 8, Moscow, 125315, Russia

**Keywords:** translocator protein, dipeptide, GD-23, anxiolytic activity, nootropic activity

## Abstract

The translocator protein (TSPO) promotes the translocation of cholesterol to the inner mitochondrial membrane and mediates steroid formation. In this study, we first report on a biological evaluation of the dipeptide GD-23 (N-carbobenzoxy-L tryptophanyl-L isoleucine amide), a structural analogue of Alpidem, the principal TSPO ligand. We show that GD-23 in a dose range of 0.05 to 0.5 mg/kg (i.p.) exhibits anxiolytic activity in the elevated plus maze test and nootropic activity in the object recognition test in scopolamine-induced amnesia in rodents. It was shown that GD-23 did not affect spontaneous locomotor activity, holding promise as a nonsedative anxiolytic agent. The anxiolytic and nootropic activities of GD-23 were abrogated by the TSPO specific ligand PK11195, which thus suggests a role for TSPO in mediating the pharmacological activity of GD-23.

## INTRODUCTION

There has been an increase in the incidence of anxiety disorders over the past several years [1]. Benzodiazepines are used to manage anxiety, binding the GABA(A) receptor α- and γ subunits and allosterically modulating GABA(A)-ergic transmission [2]. Although benzodiazepines are highly effective for the relief of anxiety, they carry the risk of negative side effects such as sedation, muscle relaxation, cognitive impairment, as well as tolerance and dependence after repeated treatment.

Promising candidates for fast-acting anxiolytic drugs free of side effects could be selective antagonists of the GABA(A) receptor α2- and α3 subunits, mediating the anxiolytic effects [2], or ligands of the translocator protein (TSPO), previously known as the peripheral benzodiazepine receptor [1].

TSPO is primarily expressed in steroid-producing cells, including central and peripheral nervous system cells, and localizes in the outer mitochondrial membrane [3]. TSPO is involved in the first steroidogenic reaction by regulating cholesterol transport into mitochondria [4]. Neurosteroids are known as endogenous GABA(A) ligands that modulate neuronal excitability, whose anxiolytic effects, for example, pregnenolone, have been described in detail [5]. TSPO and neurosteroids have been implicated in the etiology of anxiety disorders. Recent work has reported that patients suffering from symptoms of clinical anxiety have reduced levels of TSPO in blood cells and neurosteroids in the spinal fluid [6, 7]. Neurosteroids and benzodiazepines recognize different GABA(A) receptor epitopes [1], which accounts for their pharmacological properties. Numerous *in vitro *and *in vivo *studies have shown that TSPO ligands stimulate neurosteroidogenesis [5]. For this reason, TSPO ligands have emerged as fast-acting agents for the pharmacological treatment of anxiety-like disorders [5, 8].

This study extends our previous work in which we engineered a structurally similar analog of Alpidem (anxiolytic), the primary member of the pyrazolopyrimidine TSPO ligands, by synthesizing short peptides with tailored functionalities based on the chemical scaffolds of nonpeptide drug compounds [9]. The novel peptide, a substituted dipeptide amid N-carbobenzoxy- L-tryptophanyl-L-isoleucine, (GD-23) and Alpidem share structural homology with two aromatic and one aliphatic pharmacophores. GD-23 was chemically synthesized using activated N-oxysuccinimide ethers.

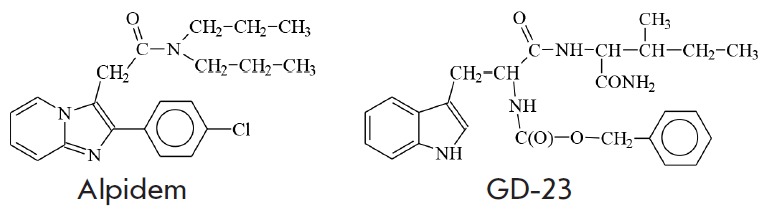



The objective of this study was to assess the pharmacological activity of GD-23 in terms of anxiolytic and nootropic effects. To confirm the role of TSPO in modulating GD-23 action, we undertook an analysis of GD-23 antagonism in the context of PK11195, the TSPO specific ligand.

## EXPERIMENTAL SECTION

**Compounds**

In a previous study of our scientific group, an amid Ncarbobenzoxy -L-tryptophanyl-L-isoleucine (GD-23) was designed [10]. The detailed data are given below: T_m_. 214–216oC, [a]*_D_*^20^ -23° (s 1; DMF); 1H NMR spectrum (DMSO-d_6_) δ:0.80 (3 H, t, C^δ^H_3_ Ile), 0.83 (3 H, d, C^γ^°H3 Ile), 1.07 and 1.44 (2 H, 2 m, C^γ^H_2_ Ile), 1.72 (1 H, m, C^β^H Ile), 2.92 and 3.11 (2 H, 2d, C^β^H Trp), 4.17 (1 H, dd, C^α^H Ile), 4.34 (1 H, m, C^α^H Trp), 4.93 and 4.98 (2 H, 2 d, -OCH_2_C_6_H_5_), 6.95–7.28 (10 H, m, -OCH_2_C_6_H_5_, indole), 7.46 (1 H, d, NH Trp), 7.77 (1 H, d, NH Ile), 7.41 and 7.13 (2 H, 2s, NH_2_ amide), 10.80 (1 H, s, NH indole). The empirical formula established as C_25_H_30_N_4_O_4_ by elementary analysis showed less than 0.4% variation from the theoretical formula. Chromatographic purity was estimated by TLC/HPLC. Scopolamine and the PK11195 inhibitor (N-butan-2-yl-1-(2-chlorophenyl)-N-methylisoquinoline- 3-carboxamide) were obtained from Sigma-Aldrich (USA).

GD-23 dipeptide and PK11195 were dissolved in a 0.05% water Tween-80 solution for intraperitoneal administration (IA) of 2 ml per kg of rat body weight. GD- 23 was injected at doses of 0.05, 0.5, 0.1, 1 and 5 mg per kg of body weight (mice and rats); PK11195 at a dose of 10 mg per kg of mouse body weight and 3 mg per kg of rat body weight [5]. Scopolamine was diluted to give 1 ml per kg of rat body weight and administered subcutaneously (SC) at 2 mg/kg. Control animals were shaminoculated with 0.05% water Tween-80 as PK11195 and GD inoculates, and with saline as scopolamine inoculates, by the same route and in the same volume.

**Animals**

Experiments were run with 107 outbred male rats (weighing 195–215 g) obtained from the animal nursery filial SCBMT Stolbovaya (Russia) and 80 CD1 male mice (weighing19–25 g) obtained from the animal breeding center at the Putschino filial of the M.M. Shemyakin and Yu.A. Ovchinnikov Institute of Bioorganic Chemistry of the RAS. The animals were housed under controlled temperature, 20–22°C, and maintained on a reversed 12:12 hour light/dark cycle, with ad libitum access to water and food. All handling and experimental procedures took place during the light phase of the cycle between 10:00 and 14:00 pm. All animals were randomly assigned into groups, balanced for weight. The animals were habituated to the test room in home cages 24 h prior to experimentation. All experimental procedures followed the guidelines for animal care of the European Community Council (86/609/CEE), and they were approved by the Bioethical Committee of the Institution (protocol № 115.09.2014).

## GD-23 ANXIOLYTIC ACTIVITY

**“Elevated plus maze test” (EPM)**

The elevated plus maze is a widely used behavioral assay for rodents [11]. The EPM apparatus was constructed of grey polyvinylchloride, consisting of two open arms connected perpendicular to two closed arms. The open arms had no walls (open arms; 65 × 5 cm). The other two arms were enclosed by opaque side walls (15 cm high). The apparatus was elevated 40 cm above ground. All arms extended from a common central platform (5 × 5 cm). The animals were placed in the center of the platform. The number of entries into the open and closed arms, the total time spent in the open and closed arms were recorded within a 5-min period. The anxiolytic activity of GD-23 was assessed using the following criteria: open arm time, open arm entries, as well as the most appropriate parameter (regardless of the activity level and time spent in the center of the apparatus) – the ratio of open arm time/entries to the total open and closed arm time/ open and closed arm entries [11].

**Experimental design**

The animals received GD-23 30 min before maze exposure. To study the antagonistic effect of GD-23 on the TSPO antagonist PK11195 was injected 30 min prior to GD-23 administration.

## NOOTRIPIC ACTIVITY OF GD-23

**Object recognition test**

The exploration of new objects in animals is based on preference for novelty and could be used to evaluate working memory [12]. The rats were housed singly in a T4 cage, similar to their home cage, lined with sawdust and were allowed 5 min to explore. Metal and glass cans filled to a volume of 0.33 ml with liquid were used as objects. Metal cans were yellow-orange, glass cans were green. All cans were tightly closed with lids. The cans used in the study were sized-matched but were visually and texturally unique. The cans were heavy enough to resist tipping and dragging inside the cage; a simple shape excluded preference for one object over an other.

The test consisted of object familiarization and test phases. During the familiarization session, the rats were exposed to two new objects in adjacent corners of the cage. Object exploration time was recorded for 4 min, followed by object removal. The break time between the phases was 3 min, while the rat was still in the same cage. During the test phase, the rat was presented with two objects in the same corners; one object was familiar from the familiarization phase, whereas the other was novel. Object exploration time was recorded for 4 min. Left and right positions of familiar and unfamiliar objects were counter-balanced across rats to avoid location bias. The objects were cleaned with ethanol to remove olfactory cues between each testing session.

Exploration of an object was defined as pointing the nose to the object at a distance of < 2 cm. For evaluation of working memory, we used the discrimination index calculated as the difference in time exploring the novel and familiar objects, expressed as the ratio of the total time spent exploring both objects [i.e., (Time Novel – Time Familiar/Time Novel + Time Familiar) × 100%], where Time Novel and Time Familiar are exploration times for novel and familiar objects, respectively .

**Experimental design**

The model of impaired cognition by scopolamine has been extensively validated to study the amnesic effects of nootropic drugs [14, 15]. GD-23 was administered 1 h before scopolamine inoculation. Thirty minutes post scopolamine inoculation, the rats were exposed to the objects. When studying the antagonistic action of GD-23 towards the TSPO inhibitor, the rats received PK11195 30 min after GD-23 administration. Thirty minutes post scopolamine inoculation, the rats were allowed to explore novel objects.

**Statistics**

Intergroup differences were analyzed with the Mann-Whitney U test with Bonferroni correction. Significance was set at p < 0.05. Data were expressed as the mean and standard deviation or median and interquartile range, as appropriate.

## RESULTS

**GD-23 exerts anxiolytic effects in EPM**

**Table 1:** GD-23 effects on CD1 mouse behavior in the EPM test

GD-23, dose, mg/kg	Open arm time, s	Closed arm time, s	Open arm entries,	Closed arm entries	Total time spent on open arms to total time spend on open and closed arms , %	Total Open arm entries to/ total open and closed arm entries , %
Control	14.25 (7.21)	232.88 (38.16)	1.88 (1.46)	7.50 (2.07)	6.06 (3.56)	19.43 (12.56)
0.1	81.88^*^ (32.40)	146.75* (43.57)	4.38^*^ (1.51)	9.25 (3.62)	36.58^*^ (15.71)	33.69 (13.17)
0.5	77.25^*^ (42.53)	198.13 (50.87)	4.13^*^ (2.36)	10.00 (3.85)	28.48^*^ (16.51)	27.79 (7.39)
1.0	20.50 (6.05)	235.25 (19.51)	1.88 (1.46)	7.75 (2.05)	8.00 (2.36)	18.03 (7.75)
5.0	14.25 (7.21)	232.88 (38.16)	1.88 (1.46)	7.50 (2.07)	6.06 (3.56)	19.43 (12.56)

Note. Data are given as a mean+(SD); each group included eight animals;*p < 0.01 versus control animals.

GD-23 dipeptide at a dose of 0.1 and 0.5 mg/kg significantly increased the open arm time (5- to 6–fold increase versus control group (p < 0.01)) and the number of open arm entries (2- to 3-fold increase versus control group (p < 0.01)) in mice. In addition, GD-23 demonstrated a 5- to 6-fold increase in the time (%) in the open arms to the total time spent in open and closed arms, which serves as an appropriate indicator of anxiolytic effects (*Table 1*). These results indicate that GD- 23 displays marked anxiolytic-like effects when administered at a dose of 0.1 or 0.5 mg/kg.

**GD-23 anxiolytic effects are dependent on TSPO interaction**

**Table 2:** The effect of the selective TSPO antagonist PK11195 on the anxiolytic effect of GD-23

Group	Open arm time, s	Closed arm time, s	Open arm entries,	Closed arm entries	Total time spent on open arms to total time spend on open and closed arms , %	Total Open arm entries to/ total open and closed arm entries , %
Control	41.50 (15.99)	131.00 (18.02)	3.13 (2.23)	8.25 (1.91)	23.87 (7.58)	25.78 (13.16)
PK11195 (10 mg/kg)	30.13 (26.82)	167.25 (13.92)^*^	3.00 (2.98)	8.63 (2.39)	14.43 (11.82)	22.20 (17.77)
GD-23 (0.5 mg/kg)	105.50^*^ (27.93)	106.88^*^ (9.51)	7.38^*^ (1.06)	7.63 (1.41)	48.99^**^ (7.87)	49.33^**^ (7.59)
PK11195 (10.0 mg/kg) + GD-23 (0.5mg/kg)	50.00^#^ (26.60)	125.13 (22.92)	5.38 (2.88)	9.38 (3.11)	27.15^##^ (11.08)	35.11^#^ (9.35)

Note. Data are given as a mean+(SD); each group included eight animals; *p < 0.01versus control animals; #p < 0.05, p < 0.01 as compared with GD-23 group (0.5 mg/kg).

Prior inoculation of PK11195, a TSPO antagonist, at a dose of 10.0 mg/kg nearly completely abrogated the anxiolytic effects of GD-23 in the EPM test (p < 0.05) (*Table 2*). The animals receiving PK11195 before GD-23 exhibited an open arm time and a percentage of time spent in open arms similar to the control. PK11195 at a dose of 10.0 mg/kg did not affect anxiety behavior; that is, the time and number of open arm entries, as well as the percentage of time and open arm entries, did not change. PK11195 seems to have antagonized GD-23 due to the competition for the same binding site. This finding indicates a role for the TSPO receptor in modulating GD-23 anxiolytic effects.

**GD-23 reverses scopolamine-induced memory impairment in rats**

**Fig. 1:**
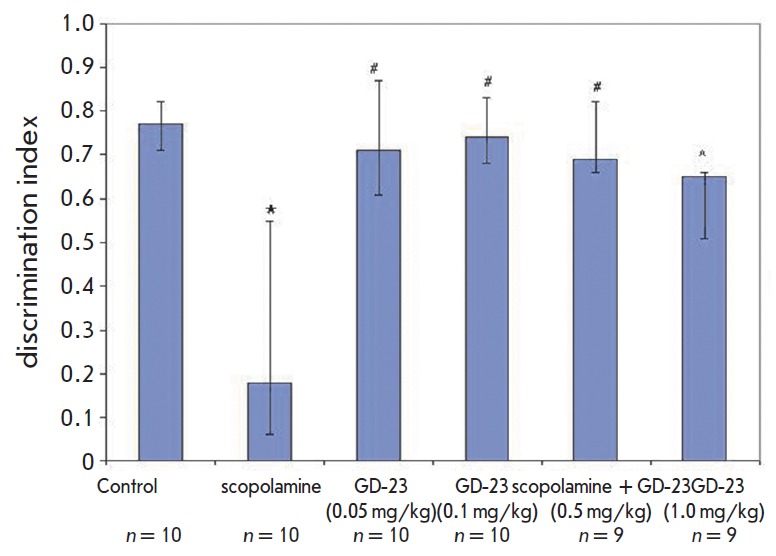
GD-23 antagonizes scopolamine-induced amnesia during exploration of novel objects. Rats received GD-23(IA) 1 h prior to scopolamine administration (0.2 μg/k, SC). Rats were allowed to explore novel objects 30 min post-scopolamine. The discrimination index showing the difference in the exploration time of a novel object and a familiar one to the total exploration time of a novel object and a familiar one during the test phase was calculated by the equation: D = (Time Novel – Time Familiar/Time Novel + Time Familiar). Data are given as median and interquartile ranges. n –number of animals in each group; ^*^p < 0.01 as compared wiith control animals, #p < 0.05 versus scopolamine animals

No intergroup differences were detected in the total object exploration time between the object familiarization and test phases (p > 0.05). Alternatively, the exploration rate was not significantly different. During the test phase, the animals took more time to explore unfamiliar objects than familiar ones (p < 0.05). Scopolamine administration significantly reduced the ability to explore objects, as shown by a 4-fold decrease in the discrimination index when compared to the control (p < 0.01, *Fig. 1*).

GD-23 at doses of 0.05, 0.1, and 0.5 mg/kg significantly alleviated scopolamine-induced amnesia with a pronounced effect at a dose of 0.1 mg/kg, whereas at a dose of 1.0 mg/kg GD-23 abolished it.

**The nootropic activity of GD-23 in amnesic rats is related to TSPO interaction**

**Table 3:** Antagonistic activity of PK11195 against the nootropic effect of GD-23 in scopolamine-induced amnesia in rats during exploration of familiar objects

Group	n	Discrimination index
Control	8	0.8 (0.75–0.9)
Scopolamine	9	0.08 (0.03–0.24)^*^
Scopolamine+GD23	10	0.66 (0.52–0.95)^#^
Scopolamine+GD23+PK11195	9	0.14 (-0.1–0.42)^^^
Scopolamine+PK11195	7	0.4 (0.23–0.44)
PK 11195	6	0.8 (0.64–0.99)

Note. Rats received GD-23 (0.1 mg/kg, IA) 1 h before scopolamine administration (0.2 mg/kg, SC). PK11195 (3 mg/kg, IA) was injected 30 min after GD-23 inoculation and 30 min prior to scopolamine exposure. Rats were allowed to explore novel objects 30 min post-scopolamine. The discrimination index showing the difference in the exploration time of a novel object and a familiar one to the total exploration time of a novel object and familiar objects during the test phase was calculated by the equation: D = (Time Novel – Time Familiar/Time Novel + Time Familiar). n –number of animals in each group,*p < 0.05 versus control animals, #p < 0.01 versus scopolamine animals, ^ – p < 0.01 as compared with “GD+scopolamine” animals. Data are given as median and interquartile ranges.

When administered, PK11195 completely antagonized the nootropic effect of GD-23 (p < 0.01). The discrimination indices (indicators of working memory) in rats treated with scopolamine alone, and scopolamine rats that had received GD-23 and PK11195, showed no significant difference (*Table 3*). PK11195 did not affect animal behavior in this test. Overall, similar to the anxiolytic affect of GD-23, the nootropic effect appears to be also associated with TSPO interaction.

## DISCUSSION

In this study, we extended our previous findings by demonstrating that the dipeptide GD-23, which was engineered based on the scaffold of Alpidem [9], the prototype compound from the imidazopyridine family of TSPO ligands, has prominent anxiolytic and nootropic effects in a dose range of 0.05–0.5 mg/kg via intraperitoneal injection. However, both activities were completely abrogated by PK11195, which indicates a role for TSPO in the pharmacological potency of GD-23.

TSPO ligands have been pursued as anxiolytic agents. Recently, a scientific group from the Beijing Institute of Pharmacology and Toxicology (Beijing, China) reported the construction of a selective TSPO ligand, YL-IPA08 [N-ethyl-N-(2-pyridinylmethyl)- 2-(3,4-ichlorophenyl)-7-methylimidazo [1,2-a] pyridine- 3-acetamide hydrochloride], capable of producing anxiolytic- and antidepressant-like effects in stress-induced mice exposed to oral doses. Moreover, this study presented data that YL-IPA08 could elevate the level of allopregnanolone in the prefrontal cortex and blood plasma of mice [8]. This compound is currently in preclinical investigation.

Japanese scientists [5] reported on the anti-anxiety and antidepressant-like effects of N-benzyl-N-ethyl- 2-(7,8-dihydro-7-methyl-8-oxo-2-phenyl-9H-purin- 9-yl)acetamide (AC-5216) upon oral administration in rats. Furthermore, AC-5216 had no myorelaxant effects nor did it affect the memory [5]. However, clinical trials of XBD173 were terminated in Phase II for lack of therapeutic efficacy [ClinicalTrials.gov identifier: NCT00108836].

There are several classes of TSPO ligands available encompassing isoquinolinecarboxamides, indoleacetamides, imidazopyridines, pyrazolopyrimidines, benzoxazepines, and phenoxyphenylacetamide derivatives [16]. Our literature search failed to identify any publications reporting the design of TSPO ligands other than those listed above. Consequently, this is believed to be the first study to report on a peptide TSPO ligand which could hold promise as a highly effective and low-toxic agent producing little or no tolerance and dependence.

GD-23 exhibits both anxiolytic and nootropic-like effects in the scopolamine induced amnesia model. These activities seem to be associated with neorosteroid stimulation, which is intrinsic to TSPO agonists. Neorosteroids such as allopregnanolone, dehydroepiandrosterone, cortisol, and corticosterone promote memory and learning; an insufficient hormone supply due to pathology or aging correlate with cognitive impairment [17]. In conclusion, in contrast to benzodiazepines, whose side effects include cognitive impairment, GD- 23 exerts nootropic effects, within dose ranges optimal for anti-anxiety action. In addition, GD-23 does not affect spontaneous activity in mice (data not shown) and thus supports the lack of sedative potential in GD-23 at the doses evaluated.

Our findings lend credence to the potential of peptide TSPO ligand GD-23 as a promising fast-acting agent for anti-anxiety therapy without the side-effects associated with benzodiazepine anxiolytics.

## Abbreviations

TSPO: translocator protein
GABA: gamma aminobutyric acid
EPM: elevated plus maze
i.p.: intraperitoneally
s.c.: subcutaneous
